# Ribosylation triggering Alzheimer’s disease-like Tau hyperphosphorylation via activation of CaMKII

**DOI:** 10.1111/acel.12355

**Published:** 2015-06-11

**Authors:** Yan Wei, Chanshuai Han, Yujing Wang, Beibei Wu, Tao Su, Ying Liu, Rongqiao He

**Affiliations:** 1 State Key Laboratory of Brain and Cognitive Science, Institute of Biophysics, Chinese Academy of SciencesBeijing, 100101, China; 2 University of Chinese Academy of SciencesBeijing, 100049, China; 3 Alzheimer’s Disease Center, Beijing Institute for Brain Disorders, Capital Medical UniversityBeijing, China; 4 Key Laboratory of Mental Health, Institute of Psychology, Chinese Academy of SciencesBeijing, 100101, China

**Keywords:** hyperphosphorylation, ribosylation, Tau protein

## Abstract

Type 2 diabetes mellitus (T2DM) is regarded as one of the serious risk factors for age-related cognitive impairment; however, a causal link between these two diseases has so far not been established. It was recently discovered that, apart from high D-glucose levels, T2DM patients also display abnormally high concentrations of uric D-ribose. Here, we show for the first time that the administration of D-ribose, the most active glycator among monosaccharides, produces high levels of advanced glycation end products (AGEs) and, importantly, triggers hyperphosphorylation of Tau in the brain of C57BL/6 mouse and neuroblastoma N2a cells. However, the administration of D-glucose showed no significant changes in Tau phosphorylation under the same experimental conditions. Crucially, suppression of AGE formation using an AGEs inhibitor (aminoguanidine) effectively prevents hyperphosphorylation of Tau protein. Further study shows AGEs resulted from ribosylation activate calcium-/calmodulin-dependent protein kinase type II (CaMKII), a key kinase responsible for Tau hyperphosphorylation. These data suggest that there is indeed a mechanistic link between ribosylation and Tau hyperphosphorylation. Targeting ribosylation by inhibiting AGE formation may be a promising therapeutic strategy to prevent Alzheimer’s disease-like Tau hyperphosphorylation and diabetic encephalopathies.

## Introduction

Type 2 diabetes mellitus (T2DM) is a metabolic disorder that currently affects over 300 million people worldwide and is now treated as a potential pandemic. T2DM is characterized by high blood D-glucose and insulin resistance. Many patients with diabetes develop chronic or long-term complications, including nerve, eye, and kidney damage (Nathan, 1993). Recent evidence suggests that patients with T2DM are at an increased risk of developing Alzheimer’s disease (AD) and that hyperinsulinemia and insulin resistance can lead to memory impairment (Janson *et al*., 2004). Previous work revealed the presence of hyperphosphorylated Tau in the brain of a T2DM animal model (Nathan, 1993; Yang *et al*., 2006; Liu *et al*., 2011). Tau hyperphosphorylation is usually considered to be an early event in the process of neurofibrillary degeneration observed in AD, which is positively correlated with the diagnosis of dementia in patients with AD (Alafuzoff *et al*., 1987; Kopke *et al*., 1993). Abnormally hyperphosphorylated Tau was discovered as the major protein subunit of paired helical filaments (PHFs) that form neurofibrillary tangles (NFTs) (Iqbal *et al*., 2010), with NFTs being a diagnostic hallmark of AD (Grundke-Iqbal *et al*., 1986; Wang & Liu, 2008). Therefore, Tau hyperphosphorylation may be also involved in cognitive impairment for T2DM patients.

Glycation occurs between a carbonyl group from a reducing sugar and a free amino group of a protein. Glycation has been known for some time to be linked to T2DM (Anonymous, 1998; Tan *et al*., 2002) and neurodegenerative diseases such as AD (Dukic-Stefanovic *et al*., 2001). The glycation end products, resulting in linkages that are not hydrolyzed by digestive enzymes, are called advanced glycation end products (AGEs) (Day *et al*., 1979; Cloos & Christgau, 2002). Li *et al*. (2012) prepared AGEs by incubation of BSA with 0.5 M D-glucose and then treated cells and rats with the AGEs. They found that AGEs can induce Tau hyperphosphorylation and impair synapse and memory through receptor for AGEs (RAGE)-mediated GSK-3 activation (Li *et al*., 2012). However, whether high levels of reducing sugar, for instance D-glucose or D-ribose, can trigger Tau hyperphosphorylation and cognitive impairment is still unclear.

D-ribose is a naturally occurring pentose monosaccharide and an essential component for energy production in cells, apart from many other important biological functions (Mauser *et al*., 1985; Keller *et al*., 1988). It can come from the foods which contain high amounts of riboflavin, such as eggs, meat, and wheat bran. Also, it can be ingested as a supplement for cardiac energetic metabolism (Gross *et al*., 1991; Murphy & Allen, 2003; Shecterle *et al*., 2010). The concentration of D-ribose is about 0.02 mm in human serum (Cai *et al*., 2005), while D-glucose is 3.9∼6.1 mm in healthy human. The concentration of D-ribose in the cerebrospinal fluid is 0.01∼0.2 mm and glucose is 0.04∼1.6 mm (Seuffer, 1977). Many years ago, Marks emphasized that pentose phosphate pathway is one of the important pathways for glucose metabolism in diabetes (Marks, 1956). Segal and coworkers observed the decrease in blood D-glucose levels following the intravenous administration of 20 g of D-ribose to three diabetic subjects (Segal *et al*., 1957). Bierman and colleagues used larger doses of D-ribose given to patients with both mild and severe diabetes and confirmed Segal’s finding (Bierman *et al*., 1959). Hammes and collaborators used benfotiamine to block three major pathways of hyperglycemia damage and prevent experimental diabetic retinopathy (Hammes *et al*., 2003). Su and colleagues found that D-ribose is significantly increased in the urine of patients with type 2 diabetes mellitus, accompanied with high levels of D-glucose (Su *et al*., 2013), suggesting that T2DM is not only related with a dysfunction in D-glucose metabolism, but also in D-ribose metabolism (Su & He, 2014). On the other hand, D-ribose is an efficient glycator (Chen *et al*., 2009, 2010; Wei *et al*., 2009) and is much more active in protein glycation than D-glucose under identical conditions. D-ribose rapidly glycates protein, resulting in aggregation of protein with high concentrations of AGEs, which are highly toxic to cells (Wei *et al*., 2012a; Lu & He, 2014). Therefore,the efficacy of D-ribose and D-glucose in protein glycation and cytotoxicity should be investigated in a comparative manner.

Calcium-/calmodulin-dependent protein kinase type II (CaMKII) is one of the most abundant Ca^2+^-regulated protein kinases in the brain and is expressed primarily in neurons (Hunter, 1987; Bulleit *et al*., 1988). In the brain, CaMKII phosphorylates a broad range of substrate proteins, including Tau, tubulin, and MAP2 (Lindwall & Cole, 1984; Baudier & Cole, 1987). CaMKII is known to be regulated by Ca^2+^-/CaM-induced autophosphorylation at multiple sites, with Thr 286/287 (K/L subunits, respectively) being the major phosphorylation sites (Miller *et al*., 1988). This autophosphorylation results in the conversion of CaMKII into its Ca^2+^-independent form, concomitant with the full activation of its total activity and the trapping of Ca^2+^/CaM (Kwiatkowski *et al*., 1988; Ikeda *et al*., 1991; Katoh & Fujisawa, 1991; Hanson *et al*., 1994; Ishida *et al*., 1996). Removal of Ca^2+^/CaM from the autophosphorylated kinase stimulates Ca^2+^-independent autophosphorylation at Thr305 and/or Thr306. This Ca^2+^-independent autophosphorylation inhibits subsequent Ca^2+^/CaM binding to CaMKII, leading to a decrease in the activity of the kinase (Hashimoto *et al*., 1987; Hanson & Schulman, 1992; Colbran, 1993). Thus, whether CaMKII participates in Tau hyperphosphorylation through CaMKII autophosphorylation of Thr286 or Thr305 remains to be elucidated.

High level of D-ribose in T2DM would induce more glycation, thus affected the brain, which might be a cause of cognitive impairment of patients (Han *et al*., 2011; Su *et al*., 2013). Investigation into the relationship between glycation and cognitive impairment should shed light on the precise mechanisms responsible for diabetic encephalopathy. However, the precise details of the mechanistic link between D-ribose/D-glucose and Tau phosphorylation remain ill-defined (Wei *et al*., 2012b). In this study, we demonstrated that D-ribose induced Tau hyperphosphorylation in C57BL/6 animal brain and neuroblastoma N2a cells, whereas no such observation was made for D-glucose. Furthermore, we showed that the activation of CaMKII was directly involved in Tau hyperphosphorylation and that inhibition of AGE formation significantly reversed D-ribose-induced Tau hyperphosphorylation by CaMKII.

## Results

### Tau hyperphosphorylation in the mouse brain after the administration of D-ribose

**Fig 1:**
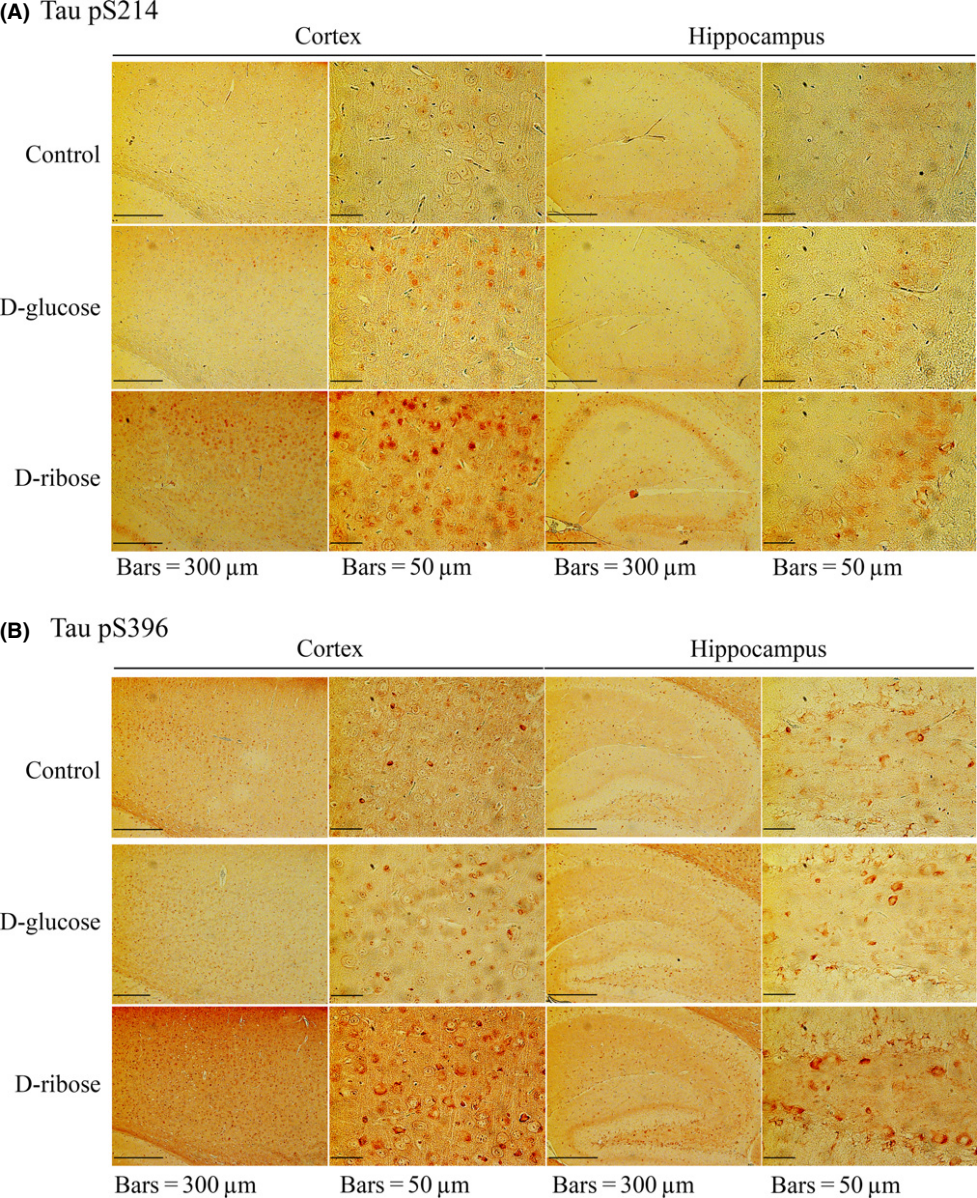
Immunohistochemistry of Tau phosphorylation in the brain of mouse injected with D-ribose and D-glucose. Mice (C57BL/6J) were injected (i.p.) with D-ribose (rib) at a dose of 2 g kg^−1^, or D-glucose (glc) at a dose of 2 g kg^−1^, or 0.9% saline (control) daily for 30 days. Tau phosphorylation in the brain was detected in both cortex and hippocampus by immunohistochemistry using anti-pSer214 of Tau (panel a) and anti-pSer396 Tau (panel b) as indicated.

As reported in our previous work, daily administration of D-ribose over the course of 30 days accelerates the AGE formation in the mouse brain and induces impairment of spatial learning and memory ability as measured in a Morris water maze test (Han *et al*., 2011). To investigate why D-ribose, but not D-glucose, induces cognitive impairment in mice, we first observed Tau hyperphosphorylation in the presence of reduced monosaccharides. As shown by immunohistochemistry, phospho-Ser214 (pSer214) of Tau was significantly increased in the mouse brain sections (Fig.1a). Furthermore, pSer396 of Tau was also observed on the brain sections (Fig.1b). In contrast, the control experiment with D-glucose only showed a small increase for both pSer214 and pSer396 signals. These results indicate that the administration of D-ribose is able to trigger hyperphosphorylation of Tau in mouse brain, whereas D-glucose cannot.

**Fig 2:**
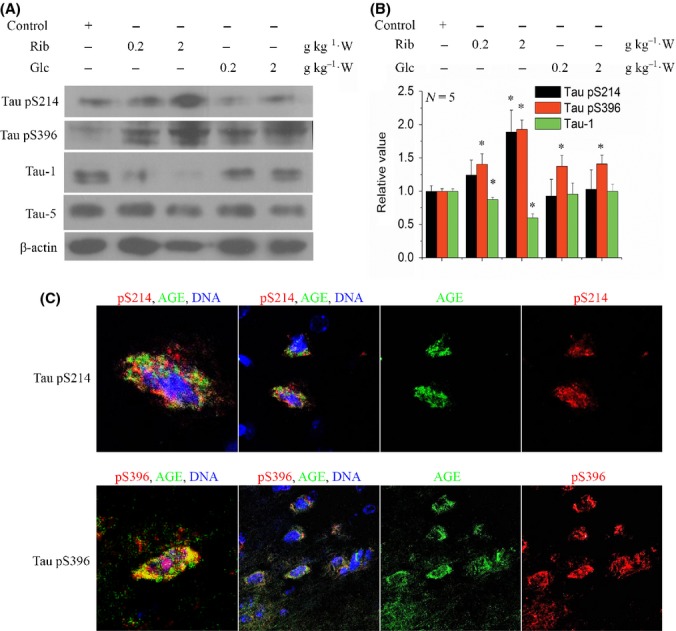
Western blotting and immunofluorescent staining of phosphorylated Tau in the mouse brain. Mice were injected (i.p.) with D-ribose or D-glucose or saline (control) daily for 30 days as indicated. Tau phosphorylation (anti-pSer214 and anti-pSer396) and nonphosphorylation (Tau-1, a monoclonal antibody against dephosphorylated Tau protein) were detected by Western blotting (20 μg brain lysate loaded). β-actin was used as a control (panel a). For each phospho-epitope, relative immunoreactive band intensities are expressed as a ratio to total Tau. For total Tau, relative values are expressed as a ratio to β-actin. Data are expressed as mean ± SD; * denotes *P* < 0.05 vs. control, respectively. Panel b shows the quantitative analyses of the data from panel a. *n* = 5. Immunofluorescent staining for Tau phosphorylation was visualized in 2 g kg^−1^ D-ribose-injected groups (panel c). Phosphorylated Tau (red), AGEs (green), and nuclei (blue) are shown.

To further demonstrate that D-ribose can trigger hyperphosphorylation of Tau, we measured the changes in Tau phosphorylation at Ser214 and Ser396 by Western blotting. As shown in Fig.2a and b, a marked increase in pSer214 and pSer396 (*P* < 0.05, *n* = 5) was observed, which occurred in a D-ribose concentration-dependent manner, even though dephosphorylated form of brain Tau protein (Tau-1) actually decreased (*P* < 0.05, *n* = 5). The addition of D-glucose, however, did not initiate any significant changes in Tau phosphorylation (*P* = 0.68194, *n* = 5). This confirms that the administration of D-ribose triggers mouse brain Tau hyperphosphorylation, whereas D-glucose does not.

Subsequent immunofluorescent experiments demonstrated the increase in phosphorylated Tau in the mouse brain (Fig.2c). However, the AGE staining signals probably did not colocalize with the signals for either pSer214 or pSer396 phosphorylation staining of Tau. There was also slight positive staining for mice injected with D-glucose (Fig. S1, Supporting information). As D-glucose is much slower in glycation reaction than D-ribose, time is also a factor in AGE formation. We have adopted a 3-month injection experiment with D-glucose. The D-glucose-injected mice did not exhibit cognitive impairment measured by Morris water maze (Fig. S2a,b, Supporting information). Long-term administration (3 months) of D-glucose induced increment of AGE production in the serum, but with slight increase in Tau phosphorylation in the brain (Fig. S2c, Supporting information).

Together, these results clearly indicate that hyperphosphorylation of brain Tau protein at Ser214 and Ser396 occurs in the presence of D-ribose, but not D-glucose.

### Direct correlation between D-ribose concentration and AGE levels in cells

**Fig 3:**
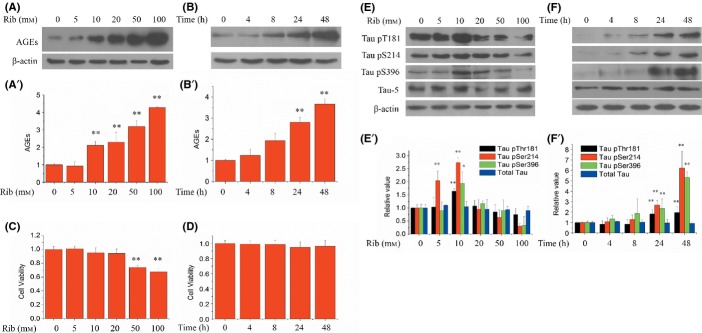
AGE formation, viability of cells, and Tau phosphorylation following the treatment of D-ribosylation in N2a cells. Different concentrations of D-ribose (final concentration 0, 5, 10, 20, 50, and 100 mm) were added to N2a cells before incubation for 24 h. AGEs were detected by Western blotting (panel a,a′, 20 μg cell lysate loaded). Cell viability was measured using CCK-8 assay (panel c). D-ribose (final concentration 10 mm) was added to N2a cells and incubated for different time lengths (0, 4, 8, 24, and 48 h), and AGE production (panel b,b′) and cell viability (panel d) were measured. *n* = 5. N2a cell lysate extracts were separated by 12% SDS-PAGE, and levels of phosphorylated Tau and AGEs were determined by Western blotting using antibodies directed at pThr181, pSer214, pSer396, or total Tau at different concentrations of D-ribose (panel e) and for different cell culture times (panel f). Panels e′ and f′ show quantitative analyses for data from panel e and f, respectively. *n* = 6. For each phospho-epitope, relative immunoreactive band intensities are expressed as ratios in comparison with total Tau. For total Tau, the relative values are expressed as a ratio to β-actin. Data are expressed as mean ± SD; * and ** denote *P *< 0.05 and *P* < 0.01 vs. control, respectively.

To investigate Tau phosphorylation in the presence of D-ribose in the culture medium, we used a mouse neuroblastoma neuro-2a cell line (N2a) as an *in vitro* model. To this end, N2a cells were incubated for 24 h with different concentrations of D-ribose (0, 5, 10, 20, 50, and 100 mm), and the yield of AGEs was measured by Western blotting (Fig.3a). The levels of AGEs in N2a cells were markedly increased (*P* < 0.01, *n* = 5) in the presence of D-ribose at the concentrations of 10 mm and higher (Fig.3a′). Also, the yields of AGEs increased with increasing incubation times when using 10 mm D-ribose (Fig.3b,b′). These data indicate that ribosylation produces high levels of AGEs in N2a cells.

Next, cell viability was assayed at different concentrations of D-ribose for different time durations (0, 4, 8, 24, and 48 h) using a CCK-8 assay. As shown in Fig.3c, the viability of N2a cells decreased in a D-ribose concentration-dependent manner. While N2a cells were treated with 10 mm D-ribose for different time durations, the cell viability displayed an observable, but insignificant decrease when compared to control cells (Fig.3d). However, when cells were incubated in D-ribose of more than 20 mm, cell viability began to decrease markedly over a period of 24 h (data not shown). D-glucose, in contrast, did not incur any significant changes in cell viability at the concentrations used (Fig. S3, Supporting information). Thus, to investigate the correlation between D-ribose and Tau phosphorylation in more detail, the concentration of 10 mm D-ribose was employed for all further experiments unless stated otherwise.

### Addition of D-ribose triggers Tau hyperphosphorylation in cells

To demonstrate the utilization of D-ribose triggering Tau hyperphosphorylation, we determined the phosphorylation of Tau at Thr181, Ser214, and Ser396 phospho-epitopes in N2a cells. Cells were exposed to different concentrations of D-ribose (0, 5, 10, 20, 50, and 100 mm) for 24 h. As shown in Fig.3e,e′, incubation in 10 mm D-ribose resulted in a significant increase in Tau phosphorylation at Thr181, Ser214, and Ser396 (*P* < 0.01, *n* = 6), while the concentration of total Tau remained unchanged. Similar to mouse brain Tau phosphorylation, pSer214 was more sensitive to D-ribose exposure (as low as 5 mm). The primary neurons showed the same result (Fig. S4, Supporting information).

Next, we treated N2a cells with 10 mm D-ribose for different time intervals (0, 4, 8, 24, and 48 h) before measuring pThr181, pSer214, and pSer396 of Tau protein. Tau phosphorylation at these three phospho-epitopes markedly increased (*P* < 0.01, *n* = 6) in direct correlation with culture time (Fig.3f,f′), while levels of total Tau did not change significantly. In contrast, 10 mm of D-glucose did not show any significant effect on Tau phosphorylation or AGE formation (Fig. S5, Supporting information). Together, these results demonstrate that the presence of D-ribose in the media induces Tau hyperphosphorylation in N2a cells under our experimental conditions.

### Impact of D-ribose on Tau kinases and phosphatases in cells

**Fig 4:**
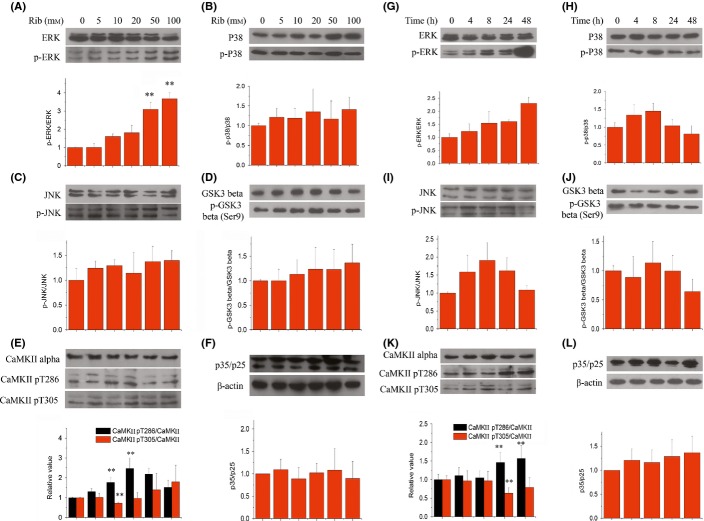
Changes in the levels of Tau kinases in N2a cells following the treatment of D-ribose. N2a cells were first treated with different concentrations of D-ribose for 24 h. Then, cell lysate extracts were separated by 12% SDS-PAGE and levels of Tau kinases were determined using antibodies directed at activated or total kinases as follows: phospho-ERK/ total ERK (panel a), phospho-p38/ total p38 (panel b), phospho-JNK/ total JNK (panel c), phospho-GSK-3β/ total GSK-3β (panel d), phospho-CaMKII/ CaMKII (panel e), and p35/25 (panel f). N2a cells were incubated with 10 mm D-ribose for different lengths of time (0, 4, 8, 24, and 48 h). N2a cell lysate extracts were separated by SDS-PAGE, and levels of Tau kinases were determined using antibodies directed at activated or total kinases as follows: phospho-ERK/ total ERK (panel g), phospho-p38/ total p38 (panel h), phospho-JNK/ total JNK (panel i), phospho-GSK-3β/ total GSK-3β (panel j), phospho-CaMKII/ CaMKII (panel k), and p35/25 (panel l). Relative immunoreactive band intensities are expressed as a ratio to control. *n* = 3. Data are expressed as mean ± SD; ** denotes *P* < 0.01 vs. control.

To clarify the mechanism of D-ribose-induced hyperphosphorylation, we examined changes in the activation of specific Tau kinases commonly involved in the regulation of Tau phosphorylation. We explored the activation patterns in N2a cells of six kinases by employing specific antibodies. The treatment of 10 mm D-ribose for 24 h resulted in a significant increase in active form of CaMKII (p-Thr286), yet simultaneously in a decrease in inactive form of CaMKII (p-Thr305) phosphorylation (Fig.4e). The enzyme activity assay supported this result (Fig. S6, Supporting information). In contrast, the p-p38/p38 ratio (Fig.4b), p-JNK/JNK ratio (Fig.4c), p-GSK-3β/GSK-3β ratio (glycogen synthase kinase-3-beta, Fig.4d), and p35/25 ratio (Fig.4f) did not exhibit any marked changes when compared to their respective controls lacking D-ribose. Also, the p-ERK/ERK ratio (Fig.4a) did not markedly change, except when 50 and 100 mm D-riboses were used. Thus, our data suggest that CaMKII is activated by D-ribose and therefore could play a major role in Tau phosphorylation.

The 10 mm D-ribose treatment experiments were also carried out for different time intervals, to confirm the results obtained as described above. Within the 24-h incubation period, a significant increase in p-CaMKII levels at Thr286 (*P* < 0.01, *n* = 3) was observed, together with a decrease at Thr305 (*P* < 0.01, *n* = 3) (Fig.4k). No significant changes were detected during the 48-h incubation for all other kinases tested (Fig4h,i,j,l), except for the p-ERK/ERK ratio (Fig.4g). Thus, this demonstrates once more that CaMKII is involved in Tau phosphorylation upon incubation with D-ribose of N2a cells.

**Fig 5:**
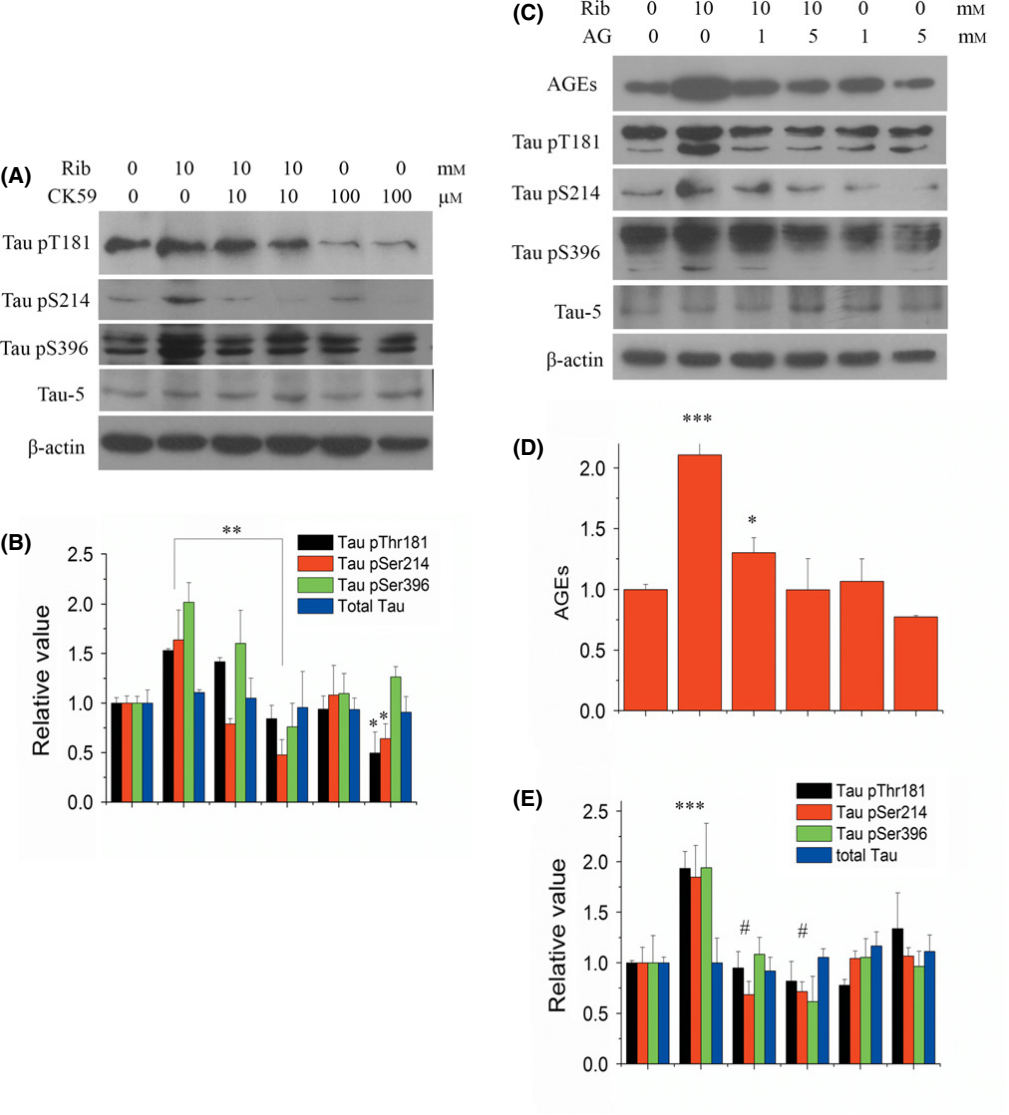
Effect of CK59 or aminoguanidine (AG) on D-ribose-induced Tau hyperphosphorylation. CK59 (10, 100 μm) was added to N2a cells together with D-ribose (10 mm), and cells were cultured for 24 h. Extracts of N2a cell lysate were separated by SDS-PAGE, and levels of Tau phosphorylation were determined using antibodies directed at Tau pThr181, pSer214, pSer396, and total Tau (panel a). Data from panel (a) were quantitatively analyzed (panel b). AG (1, 5 mm) was added with D-ribose (10 mm) to N2a cells and cultured for 24 h. N2a cell lysate extracts were separated by SDS-PAGE, and levels of Tau phosphorylation and AGEs were determined using antibodies directed at Tau pThr181, pSer214, pSer396, total Tau (Tau-5), or AGEs (panel c). The same data from panel (c) were quantitatively analyzed (panels d, e). For each phospho-epitope, relative immunoreactive band intensities are expressed as a ratio to total Tau. For total Tau and AGEs, relative values are expressed as the ratio to β-actin. *n* = 3. Data are expressed as mean ± SD; *, ** and *** denote *P* < 0.05, *P* < 0.01 and *P* < 0.001 vs. control, respectively. # denotes *P* < 0.05 vs. D-ribose treatment.

To add further proof to our assumption that CaMKII plays a major role in D-ribose-induced Tau phosphorylation, we inhibited the activation of CaMKII using a specific inhibitor CK59 (Konstantopoulos *et al*., 2007) and tested for suppression of Tau phosphorylation. Relative to the levels in the absence of the inhibitor, pSer214 levels were found to be significantly reduced in the presence of 10 μm CK59 (Fig.5a,b). Hundred μm CK59 significantly alleviated the levels of Tau phosphorylation for pThr181, pSer214, and pSer396 (*P* < 0.05, *n* = 3), among which pSer214 levels displayed the largest decrease (*P* < 0.01, *n* = 3). In the absence of D-ribose, CK59 also suppressed pThr181 and pSer214 phosphorylation, but not pSer396. Again, these data verify that CaMKII plays a major role in Tau hyperphosphorylation in the presence of D-ribose.

The levels of Tau phosphorylation are not only regulated by kinases, but also by phosphatases. We therefore examined the levels of protein phosphatase 2 (PP2A), the main Tau phosphatases. Tau is dephosphorylated by PP1, PP2A, PP2B, and PP5, with PP2A showing the strongest ability to do so (Wei *et al*., 2012b). Western blotting results indicate that at the level of protein expression, there is no significant difference for PP2A between D-ribose-treated N2a cells and control cells (Fig. S7, Supporting information). Therefore, D-ribose appears to have no impact on the levels of PP2A phosphatase; thus, no clear correlation is evident between Tau hyperphosphorylation and changes in PP2A phosphatase levels, although some changes in phospho-Tyr307/PP2A were observed at higher concentrations of D-ribose (Fig. S7a,a′, Supporting information). The methylation level of PP2A showed little change in cells after D-ribose treatment, indicating methylation of PP2A did not involve in Tau hyperphosphorylation. The PP2A activity assay confirmed that D-ribose did not have an impact on PP2A activity (Fig. S8, Supporting information).

### Ribosylated AGE is essential for Tau phosphorylation in N2a cells

Based on the results described above, it is clear that in N2a cells, D-ribose triggers the production of AGEs, followed by the induction of Tau hyperphosphorylation. Thus, whether D-ribose or ribosylated AGEs directly trigger Tau phosphorylation remains to be investigated. To this end, we employed aminoguanidine (AG), an effective inhibitor for the production of AGEs to answer this question. In the presence of AG, the levels of AGEs decreased significantly (*P* < 0.01, *n* = 4), to about half compared to that for the control (Fig.5c,d). Five mm AG completely alleviated the increase in AGEs observed in the presence of D-ribose in N2a cells. Importantly, the addition of AG not only reduced the production of AGEs, but resulted in a significant decline in signal for three Tau phospho-epitopes (pThr181, pSer214, and pSer396) as observed during incubation with D-ribose (*P* < 0.05, *n* = 3), compared to the sample without inhibitor (Fig.5c,e). Similarly, upon the addition of AG, the highest reduction was observed for the Ser214 phosphorylation site of Tau (*P* < 0.01, *n* = 3) when compared to the sample without inhibitor. AG can also suppress the activation of CaMKII under ribose treatment (Fig. S9, Supporting information). Thus, our results suggest that Tau hyperphosphorylation was a result of ribosylated AGEs, rather than due to a direct reaction involving D-ribose.

## Discussion

In the work presented here, we investigated the possible mechanistic link between T2DM and age-related cognitive impairment. Type 2 diabetes mellitus has recently been shown to be one of the major risk factors for developing AD (Qu *et al*., 2012). To understand the causal relationship between these two diseases, we investigated whether there is a mechanistic link between two key observations, namely elevated levels of D-ribose in T2DM on the one hand (Su & He, 2014) and Tau hyperphosphorylation, an early event in the development of age-related dementia during progression of AD, on the other. Using both *in vitro* and *in vivo* model systems, we examined the link between D-ribose levels and Tau hyperphosphorylation in a mouse model, the correlation between AGE aggregates and D-ribose levels *in vitro*, the levels of Tau phosphorylation in response to increased levels of D-ribose, the impact of D-ribose on the activity of Tau-associated kinases, and changes in Tau phosphorylation levels through the inhibition of ribosylation with AG.

In summary, we could show that ribosylation, as measured by the formation of AGEs in response to elevated D-ribose levels, promotes Tau hyperphosphorylation in both mouse brain and a N2a cell line via the activation of Tau-specific kinase CaMKII, ultimately resulting in dysfunction of neural cells, and that such D-ribose-induced hyperphosphorylation of Tau could be prevented in the presence of an inhibitor of AGE formation.

It was shown previously that the direct causes of Tau phosphorylation are the overactivation of protein kinases and/or inactivation of protein phosphatases (PP) (Iqbal *et al*., 2005). Here, we show that the CaMKII (but not GSK-3β) was activated in the presence of 10 mm D-ribose, indicating that CaMKII has the potential to play an important role in the phosphorylation of Tau in the presence of ribosylated AGEs. This mechanism of Tau hyperphosphorylation differs from that induced by glucosylated AGEs, where GSK-3 is activated instead (Li *et al*., 2012), and ribosylated and glucosylated AGEs enhance Tau phosphorylation using different kinase pathways. Note that D-glucose does not show any direct effect on Tau phosphorylation except for glucosylated AGEs. In our experimental setup, in contrast, these other kinases (ERK1/2, JNK, p38, p25/35, and GSK-3) did not appear to play any role in Tau hyperphosphorylation. Therefore, the increase in Tau phosphorylation is most likely a result of the activation of CaMKII in N2a cells. CaMKII is thought to be an important mediator of learning and memory. As CaMKII is implicated in long-term potentiation (LTP), D-ribose-induced activation of CaMKII may influence LTP of cells. The precise role of CaMKII for the formation of AGEs will need to be investigated in more detail in future studies, using both *in vitro* and *in vivo* model systems.

Among the phosphorylation sites investigated here (Ser214, Thr181, and Ser396), Ser214 was the most sensitive site to D-ribose exposure. Its phosphorylation status changed in a D-ribose concentration-dependent manner. In agreement with our findings, earlier work presented evidence that the phosphorylation at Ser214 is upregulated in AD-affected brains (Sadik *et al*., 2009). Furthermore, this phosphorylation site has been described as a specific marker for AD earlier (Ksiezak-Reding *et al*., 2003). Also, Ser214, together with Thr212, is located in the consensus phosphorylation sequence of CaMKII (RXXS/T), and non-fetal-type phosphorylation sites of Tau, which may produce PHF-Tau (Yoshimura *et al*., 2003). Yang *et al*. found that Ser214 is significantly hyperphosphorylated in a T2DM rat model (Yang *et al*., 2010). In this work, Ser214 may be directly responsible for the changes in the levels of D-ribose as well as ribosylated AGEs. Altogether, these results suggest that the CaMKII-mediated phosphorylation of Tau (Ser214) is critically involved in the pathogenesis of both age-related cognitive impairment and diabetic encephalopathy (Sima, 2010). But it should also be noted that D-ribose not only induced the phosphorylation of Tau but also other proteins in the cell, for instance CaMKII.

We choose aminoguanidine (AG) as an inhibitor of ribosylation and, as expected, observed the inhibition of accumulation of ribosylated AGE. Interestingly, it also suppressed Tau phosphorylation, in particular at the site pSer214, suggesting that AG can inhibit glycation, which would then reduce AGE levels and thus indirectly reduce the signals associated with Tau phosphorylation.

As mentioned above, D-ribose produces high levels of AGEs, which in turn triggers hyperphosphorylation of Tau. Therefore, Tau phosphorylation is a direct result of ribosylated AGEs and only indirectly caused by D-ribose. This viewpoint is based on our observations as described in the results section and those of others, as cited above. Tau hyperphosphorylation is observed in the presence of D-ribose following the formation of AGEs in N2a cells. Tau phosphorylation markedly decreased upon inhibition of AGE formation in the presence of the inhibitor AG.

**Fig 6:**
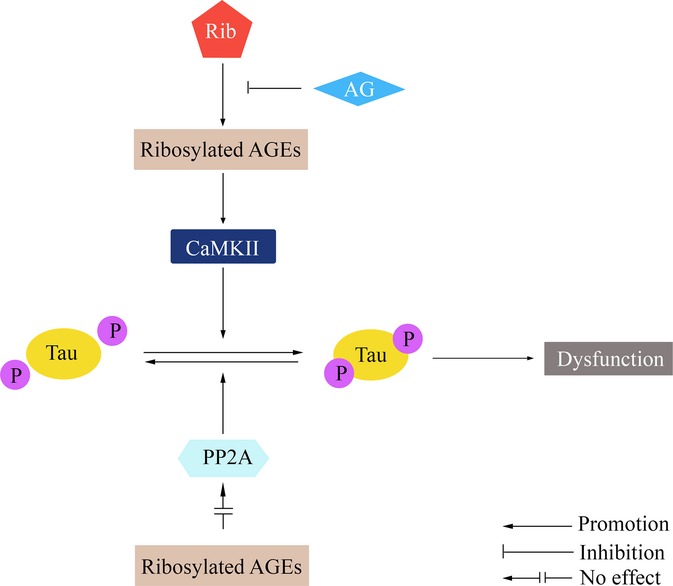
The proposed mechanism of D-ribosylation-induced Tau hyperphosphorylation.

Figure6 depicts the process of Tau hyperphosphorylation in the presence of D-ribose. D-ribose, as the most active glycator, reacts quickly with cellular proteins and produces higher yields of AGEs compared with D-glucose. The ribosylated forms of AGEs then activate CaMKII, which in turn catalyzes the phosphorylation of Tau protein. Accumulation of hyperphosphorylated Tau finally results in cell dysfunction, usually ending in cell death. At least in N2a cells, this procedure can be suppressed *in vitro* by adding AG, resulting in the inhibition of AGE production.

When looking at the correlation between cell viability and Tau phosphorylation, we found that the viability of N2a cells begin to decrease at a concentration of 10 mm D-ribose, albeit only observably compared to controls, while Tau was hyperphosphorylated at the same concentration. Also, the AGEs level measured by Western blot showed to increase from 10 mm. This concentration of D-ribose (10 mm) seems to be the turning point of cell state. As Tau hyperphosphorylation is the early event in AD development, our results could be utilized to build a cellular model to simulate the initiation of Tau hyperphosphorylation by D-ribose. We not only found Tau hyperphosphorylation in D-ribose-treated cells and animals, but also linked it with glycation. These results should encourage future investigations into the details of the link between glycation and Tau modification.

The administration of 5–10 mm D-ribose induces the increase in Tau phosphorylation, but phosphorylated Tau did not increase in the presence of 20–100 mm D-ribose. One possible explanation for this observation is that the viability of N2a cells under these conditions suffered, and the cells stopped responding to D-ribose at such high concentrations. As reported, 20 mm D-ribose induced astrocytic inflammation (Han *et al*., 2014). However, the p-ERK/ERK ratio did not change drastically, except for D-ribose concentrations higher or equal to 50 mm. In the absence of D-ribose, the addition of 100 μm CK59 also suppressed the levels of pThr181 and pSer214, which became lower than the normal control except for pSer396. This suggests that CaMKII has a background activity under physiological conditions, and it is further inactivated in the presence of CK59.

In the D-ribose-treated mouse brain, there seems no colocalization of the signal for either pS214 or pS396 phosphorylation staining of Tau and the AGE staining. This may be because D-ribose induced AGEs formation, then CaMKII activation, and finally Tau hyperphosphorylation, as we observed in N2a cells and depicted in Fig.6. So AGEs probably did not trigger Tau hyperphosphorylation directly.

In our opinion, AGEs derived from D-glucose and D-ribose should be different in the quantities and characteristics as AGEs derived from ribose are more cytotoxic than those derived from glucose, and the difference of the molecular structures between D-ribose and D-glucose. However, the detailed different properties need more investigation.

In conclusion, using *in vitro* and *in vivo* evidence, we established a mechanistic link between elevated levels of D-ribose and hyperphosphorylated Tau, the molecular basis for the appearance of neurofibrils, which have been shown previously to be directly linked to the development of age-related dementia during progression of AD. This work represents evidence that there is indeed a biochemical link between these two devastating illnesses that affect an ever-increasing number of people worldwide, adding strength and depth to the overall idea that AGEs may play a role in AD.

Therefore, targeting glycation and AGEs may present a promising therapeutic strategy to prevent AD-like Tau hyperphosphorylation and thus diabetic encephalopathy.

## Experimental procedures

### Antibodies

Antibodies utilized in this study were directed against phosphorylated Tau at the following epitopes: pThr181 (1:1000 dilution; Signalway antibody, College Park, Maryland, USA), pSer214 (1:2000; Invitrogen, Grand Island, NY, USA), pSer396 (1:2000; Invitrogen), and Tau-1 (anti-nonphosphorylated Ser199/202 of Tau, 1:4000; Millipore, Billerica, MA, USA). Total Tau was detected using Tau-5 (1:2000; Millipore). Monoclonal antibodies against AGE (1:2000, 6D12; Wako, Osaka, Japan) and β-actin (1:5000; Santa Cruz, Dallas, Texas, USA) were employed.

Changes in Tau kinase levels were examined using phospho-calcium-/calmodulin-dependent protein kinases II (CaMKII, Thr305, 1:1000; Millipore) and p25/p35 (1:2000; Beyotime, Haimen, China), and the following antibodies were purchased from Cell Signaling: GSK-3β (1:2000) and phospho-GSK-3β (Ser9, 1:2000), p44/42 MAPK (ERK 1/2, 1:2000) and phospho-ERK/1/2 (Thr202/Tyr204, 1:2000), p38 MAPK (1:1000) and phospho-p38 MAPK (Thr180/Tyr182, 1:1000), SAPK/JNK (1:1000) and phospho-SAPK-JNK (Thr183/Tyr185, 1:1000), and total CaMKII (1:1000) and phospho-CaMKII (Thr286, 1:1000).

Changes in Tau phosphatases were examined using PP2A-C subunit (1:1000; Cell Signaling, Danvers, MA, USA), phospho-PP2A (Tyr307, 1:2000; Abcam, Cambridge, MA, USA), and PP2A (methyl Leu309, 1:1000; Abcam). The Western blotting of dilution experiments was shown in Fig. S10 (Supporting information).

### Animal handling and sample collection

Male C57BL/6J mice (8–10 weeks) were obtained from Vital River Laboratory Animal Technology Co. Ltd. (Beijing, China). After 1 week of acclimatization, mice were randomly divided into five groups and, for 30 days, received either daily intraperitoneal injections of D-ribose, at doses of 0.2 or 2 g/kg, D-glucose at identical doses, or 0.9% saline (controls). There were 12 mice for each group. All mice were maintained in approved animal facilities under pathogen-free conditions. After behavioral testing, mice were sacrificed and their brains were immediately dissected out, homogenized in lysis buffer (Beyotime), and then centrifuged, to yield both supernatants for Western blots, and samples for fixation in 4% paraformaldehyde to be used for immunohistochemistry experiments. All animal experiments were carried out in accordance with the Guidelines for the Care and Use of Laboratory Animals of the National Institute of Health and were approved by the Biological Research Ethics Committee, Institute of Biophysics, Chinese Academy of Sciences (approval ID SYXK 2010-128).

### Immunohistochemistry and immunofluorescent staining

Immunohistochemistry experiments were performed as previously described (Sasaki *et al*., 2001). In brief, mice brains were immersed in 4% paraformaldehyde for 48 h immediately after they were dissected out. After fixation, brains were embedded in paraffin blocks. Sections of five micrometer thickness were processed for immunohistochemical analysis. Deparaffinized and hydrated sections were incubated in target retrieval solution at 95 °C for 30 min for the enhancement of immunoreactivity, then permeabilized with 0.3% H_2_O_2_ in absolute methanol for 10 min to block endogenous peroxidase, and incubated in 10% normal goat serum in PBS at room temperature for 30 min. The specimens were incubated overnight at 4 °C in anti-Tau pSer214 or pSer396 antibody solution diluted in PBS. After three washes in PBS, sections were incubated with biotin-labeled secondary antibodies (37 °C, 1 h). The proteins were detected using horseradish peroxidase-labeled antibodies (37 °C, 1 h), and staining was visualized with an AEC system (Nikon Optical, Tokyo, Japan).

Immunofluorescent staining was performed as described previously (Planel *et al*., 2004). In brief, after deparafinization, hydration, and enhancement of immunoreactivity, sections were incubated in 10% normal goat serum in PBS at room temperature for 30 min and probed overnight at 4 °C with primary antibodies diluted in PBS. Bonded antibodies were visualized with Alexa 488-conjugated anti-mouse IgG (Invitrogen), and cell nuclei were stained with the DNA-specific fluorescent reagent Hoechst 33258. Immunolabeled tissues were observed under an Olympus FV500 laser scanning confocal microscope (Olympus, Tokyo, Japan).

### Cell culture

Mouse neuroblastoma cell line Neuro 2A (N2a) cells (obtained from Cell Resource Center, Beijing, Amresco, Solon, OH, China) were cultured in Dulbecco’s modified Eagle’s medium supplemented with 100 IU mL^−1^ penicillin and 100 μg mL^−1^ streptomycin and 10% fetal bovine serum at 37 °C in a humidified incubator maintained at 5% CO_2_. Cells were grown to 70–80% confluence in 60-mm-diameter dishes and fed every fourth day. Cells were incubated with D-ribose (Amresco, USA) at the concentrations of 0, 5, 10, 20, 50, and 100 mm for 24 h, or 10 mm for 0, 4, 8, 24, and 48 h, or cultured with or without AG or CK59 for 24 h. Cells were then collected for cell viability testing and preparation of cellular extracts for Western blots.

### Assay of cell viability

Cell viability was assessed using a cell counting kit-8 (CCK-8; Beyotime). N2a cells were seeded into 96-well plates at a concentration of 10^4^ cells per well and exposed to D-ribose after 24 h, except for the control. For serial dilution tests, the final concentrations of D-ribose used were 0, 5, 10, 20, 50, and 100 mm and the CCK-8 reagent was added 24 h later. For testing of different incubation times, the final concentration of D-ribose was 10 mm and the CCK-8 reagent was added at 0, 4, 8, 24, and 48 h after adding D-ribose. Plates were incubated at 37 °C for 1 h and the absorbance recorded at 450 nm.

### Cell lysate protein extract preparation

In the preparation of cell lysate protein extract for D-ribose exposure, N2a cells were transferred into 6-well plates at a concentration of 3 × 10^5^ cells per well. D-ribose treatment was performed 24 h later. After indicated periods of time, the growth medium was removed by aspiration, and each well was washed two times with 1 mL of PBS. The cells were harvested in ice-cold WIP buffer (Beijing Cellchip Biotechnology Co., Ltd., Beijing, China) containing phosphatase and protease inhibitor (1 mm PMSF). The samples were then centrifuged at 20 000 *g* at 4 °C for 15 min, and the supernatants were collected and analyzed for protein content using a bicinchoninate acid (BCA) assay kit (Thermo Fisher Scientific, Waltham, MA, USA). Samples were stored at −80 °C until further use for immunoblotting analysis.

### Western blotting

Protein samples of brain tissues and cell lysate were mixed with 5 × SDS-PAGE Sample Buffer (Genstar, China) and boiled for 10 min. Expression levels of phosphorylated Tau, total Tau, and Tau kinases and phosphatases were determined using SDS-PAGE coupled with Western blotting. Cell lysate extracts containing ∼ 30 μg protein were separated on a 12% SDS-PAGE and then transferred onto PVDF membranes (Millipore). Nonspecific binding sites of the membranes were blocked with 5% nonfat milk in PBS containing 0.1% Tween-20 (PBST, pH 7.4) for 1 h at room temperature. The membranes were then incubated overnight at 4 °C with antibodies directed against total Tau, phosphorylated Tau, specific Tau kinase or phosphatase, AGEs, or β-actin. The following day, membranes were washed three times and then incubated for 1 h at room temperature with a horseradish peroxidase-linked secondary anti-mouse or anti-rabbit antibody (1:5000 dilution; ZSGB-BIO, Beijing, China). The membranes were washed again three times with PBST, and the immunoreactive bands were then visualized using enhanced chemiluminescence detection reagents (Applygen, Beijing, China). The immunoreactive bands were visualized after exposure of the membranes to Kodak X-ray film and quantified by Quantity One 1D analysis software 4.5.2 (Bio-Rad, Hercules, California, USA).

### Data analysis

All values reported represent means ± standard deviation (SE), except where otherwise indicated. Data were analyzed employing Adobe Photoshop 8.0.1, Adobe Syterm, San Jose, CA, USA, Origin 8.0, OriginLab, Northampton, MA, USA, and Microsoft Excel 2010 statistical software, Microsoft, Redmond, WA, USA. When there were multiple bands present, we used all bands for statistical analysis. For animal experiment, ‘*n*’ means the number of mice used. For cell experiments, ‘*n*’ means independent experiments. Differences between experimental groups were considered significant at a probability < 0.05 on a two-tailed test; * and ** denote *P* < 0.05 and *P* < 0.01 vs. control, respectively.

## Supporting Information

**Fig. S1** Immunofluorescent staining of AGEs and phosphorylated Tau in the mouse brain. **Fig. S2** Performance of mice injected with D-glucose in the Morris water maze test and Western blotting of phosphorylated Tau in the mouse brain. **Fig. S3** Viability of cells treated with D-glucose. **Fig. S4** Tau phosphorylation following the treatment of ribosylation in rat primary neurons. **Fig. S5** AGEs formation and Tau phosphorylation following the treatment of D-glucose in N2a cells. **Fig. S6** Activity assay of CaMKII. **Fig. S7** Effect of D-ribose on Tau phosphatases in N2a cells following the treatment of D-ribose. **Fig. S8** PP2A activity assay. **Fig. S9** Effect of aminoguanidine (AG) on CaMKII activity in N2a cells. **Fig. S10** Dilution experiment for representive antibodies.

** **
