# Supplementary figures and images for: Ribosylation triggering Alzheimer’s disease-like Tau hyperphosphorylation via activation of CaMKII
Source: Aging Cell. 2015 Jun 11;14(5):754–63. doi: 10.1111/acel.12355 (PMC4568963; doi:10.1111/acel.12355)

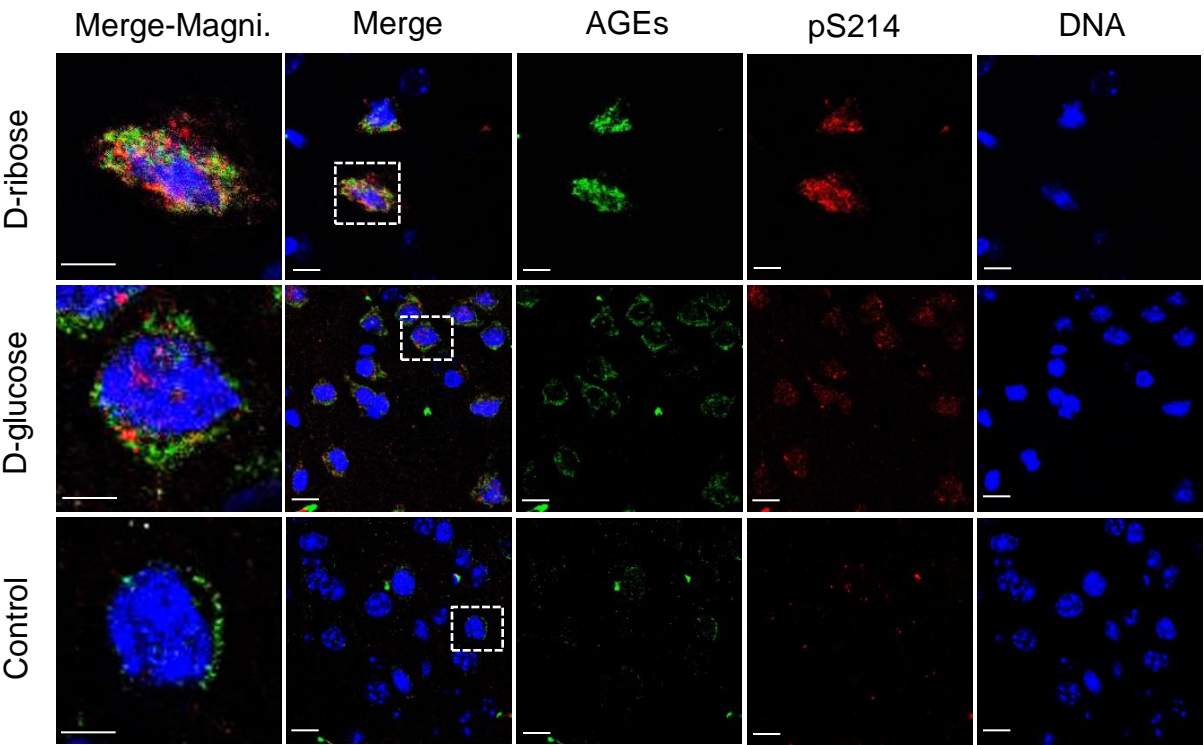

Bars=20  $\mu$ m

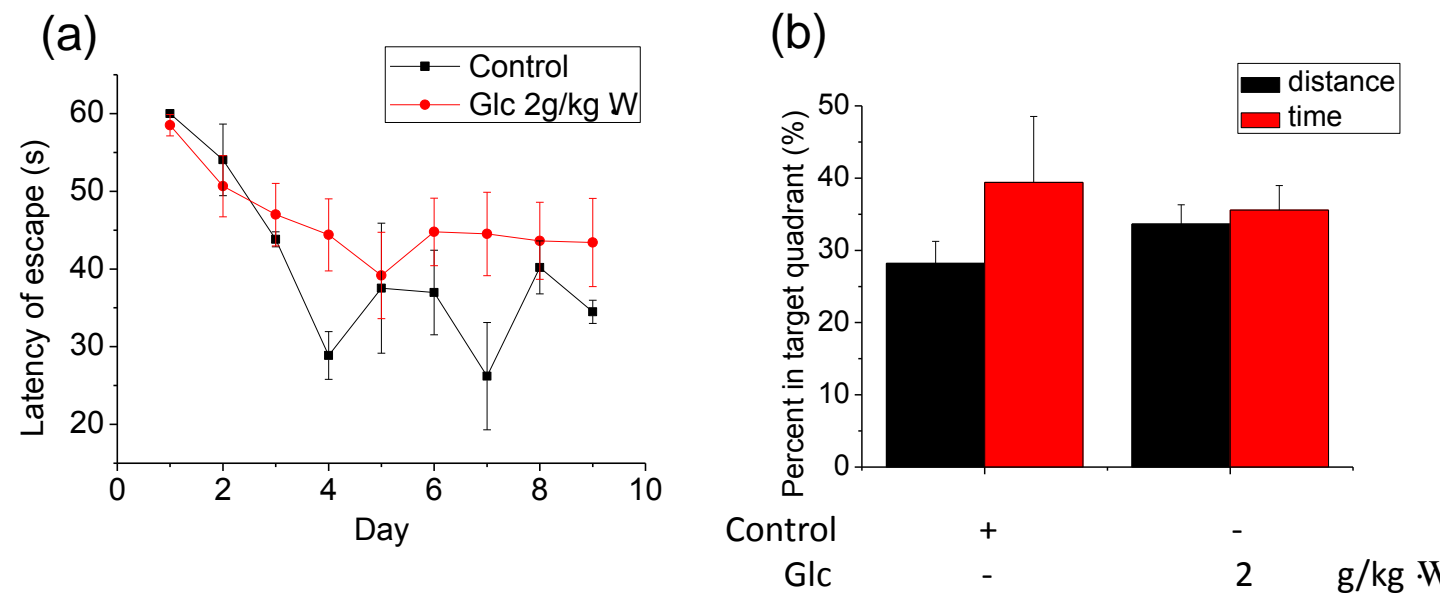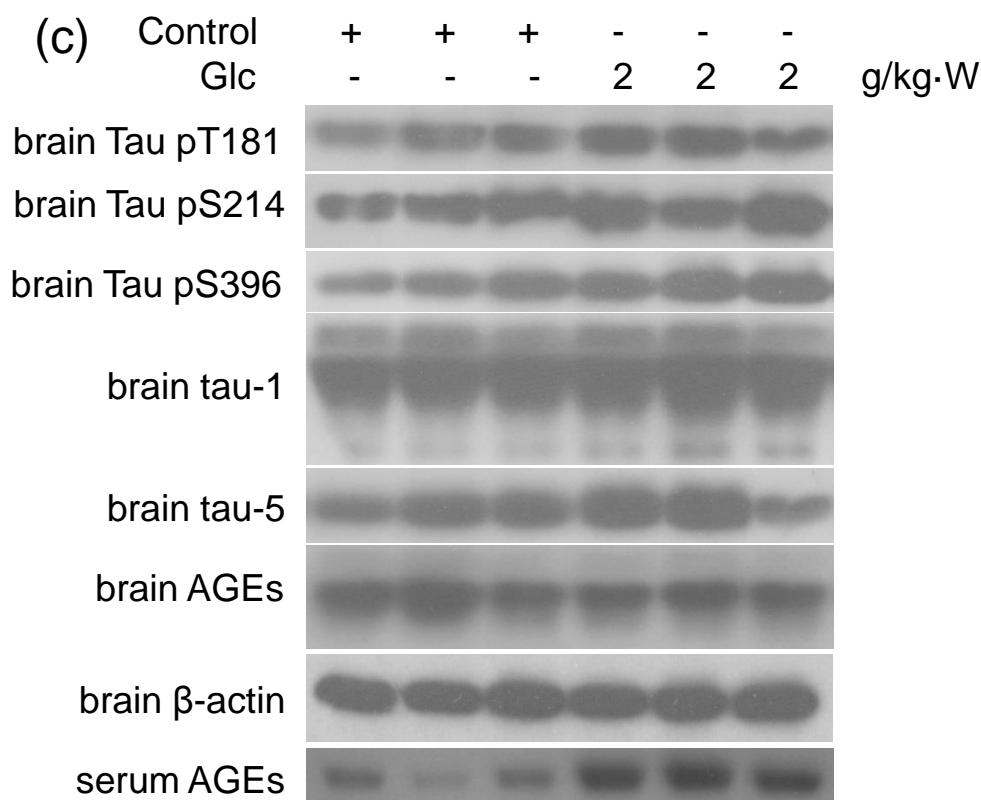

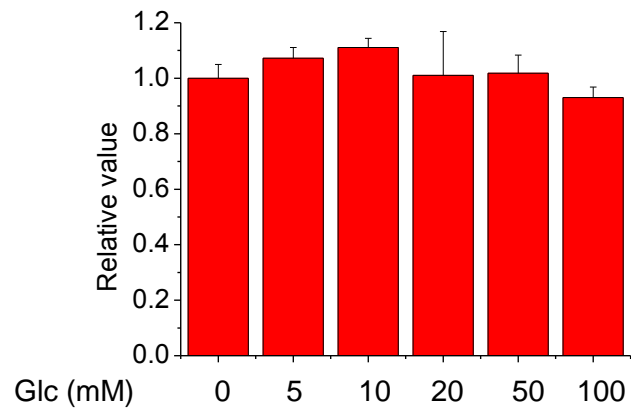

Supplementary Figure 4

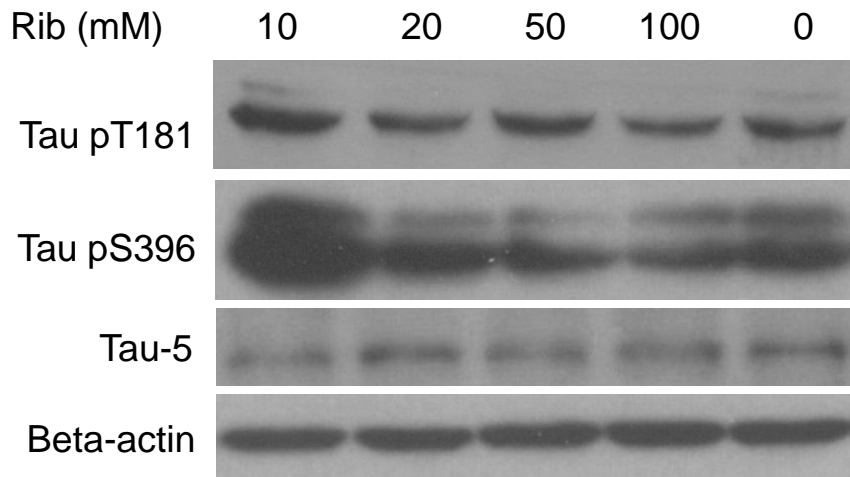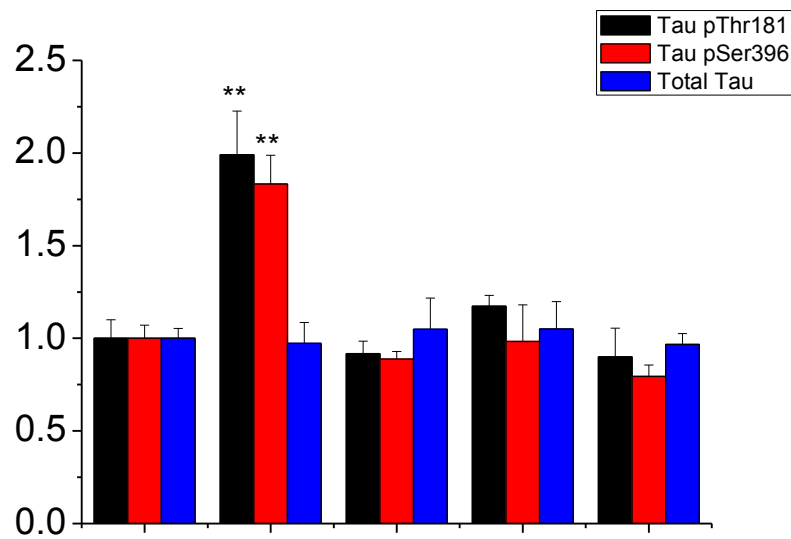

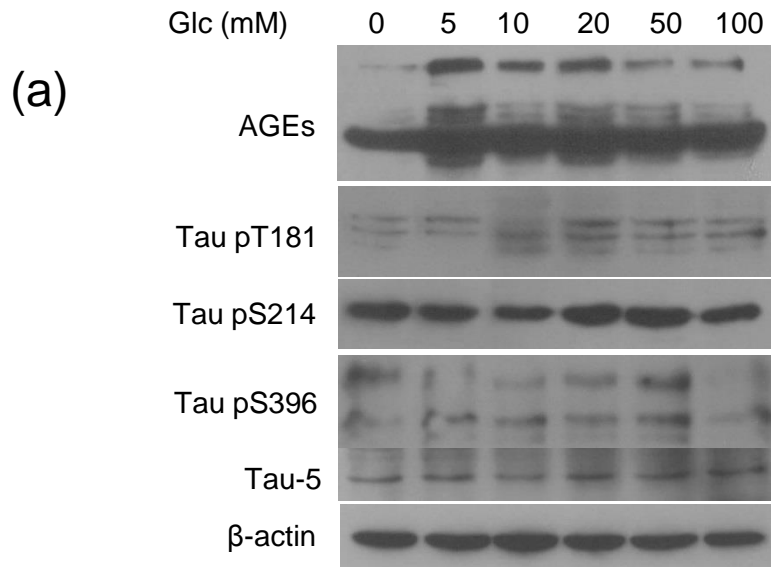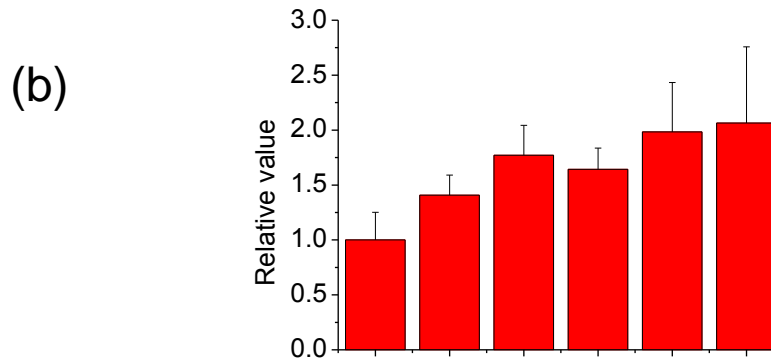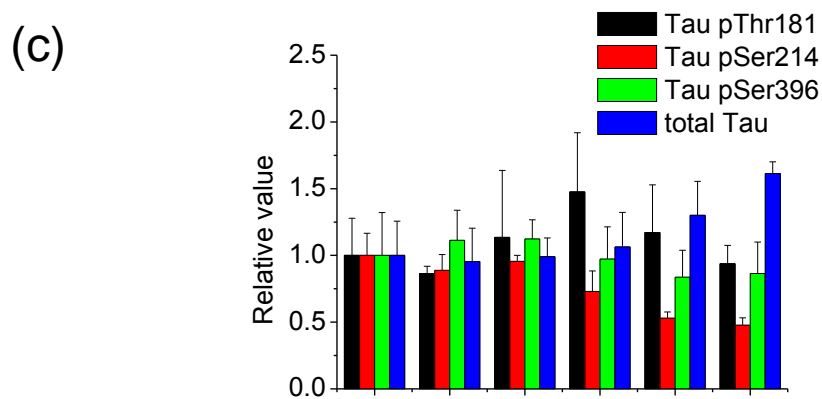

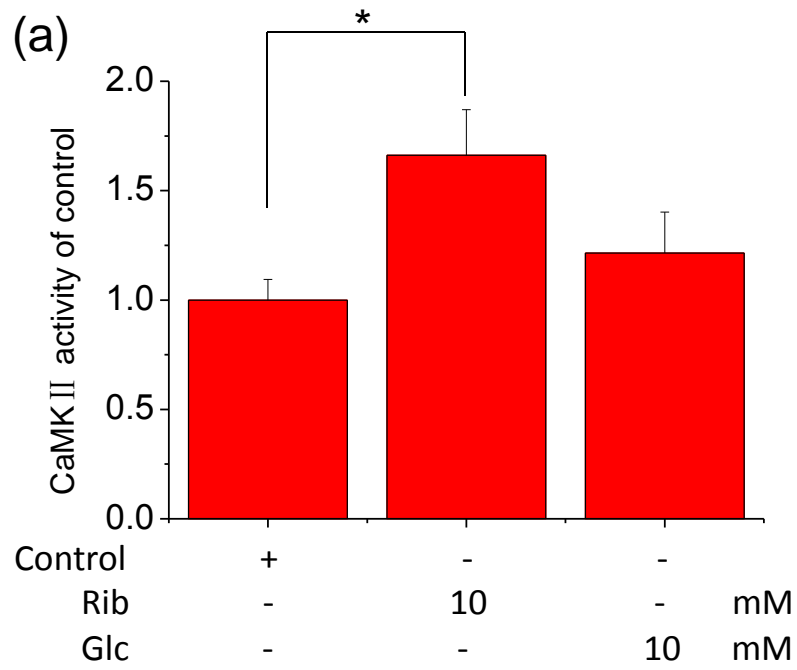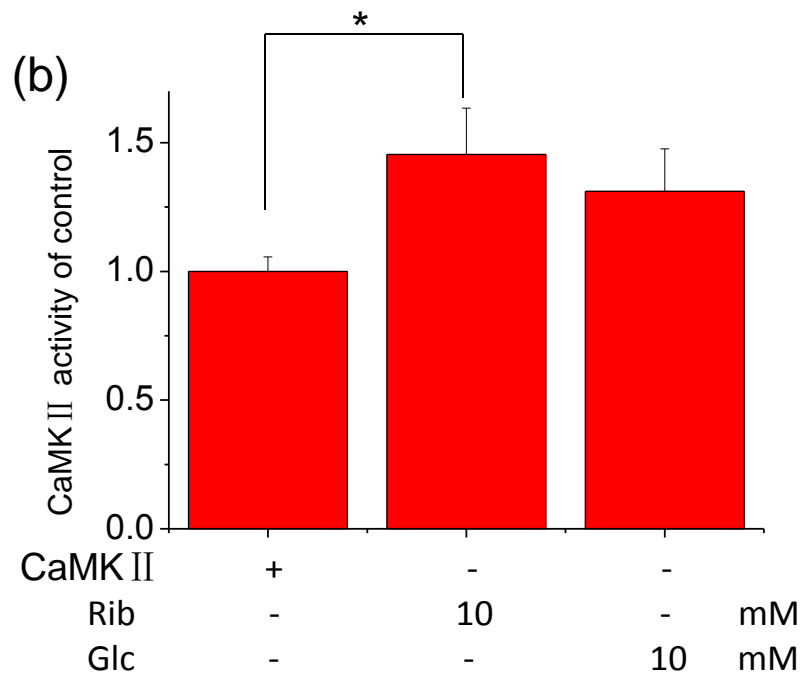

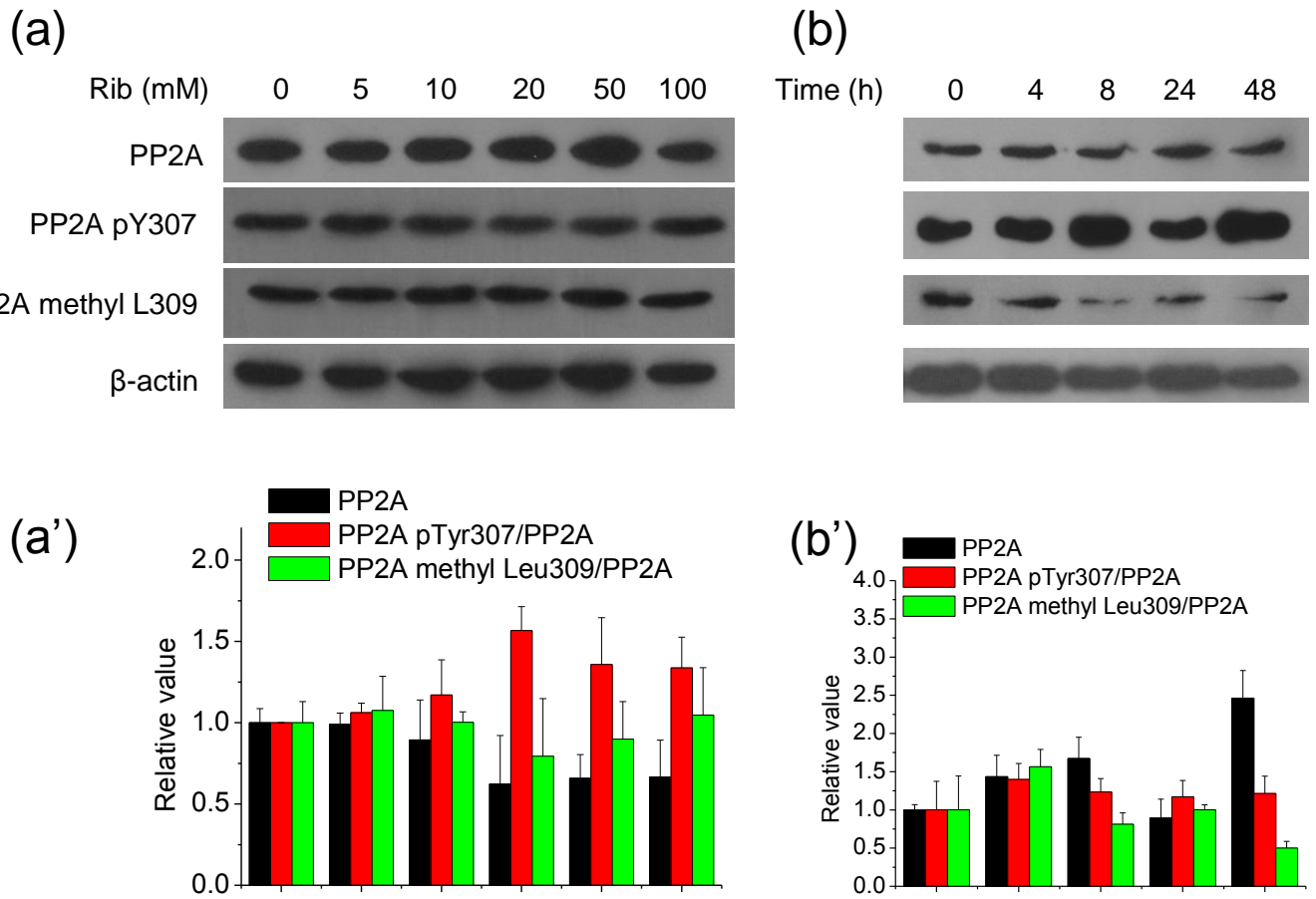

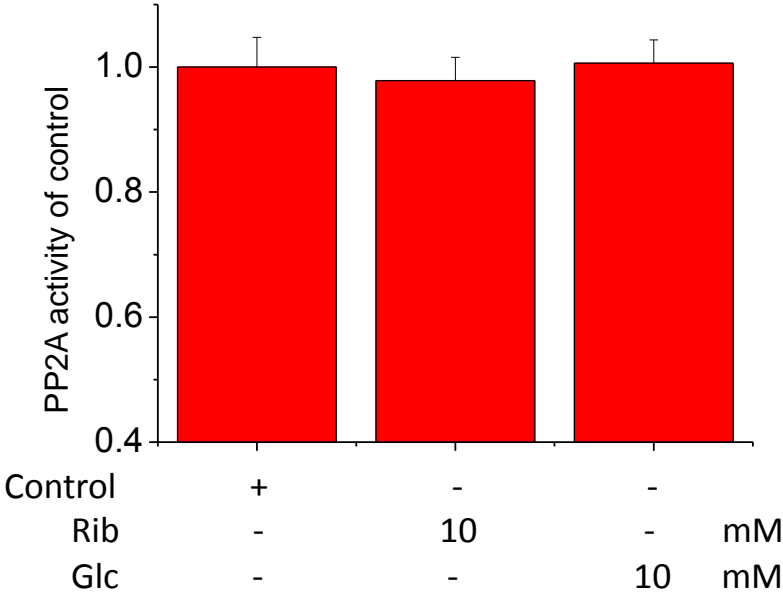

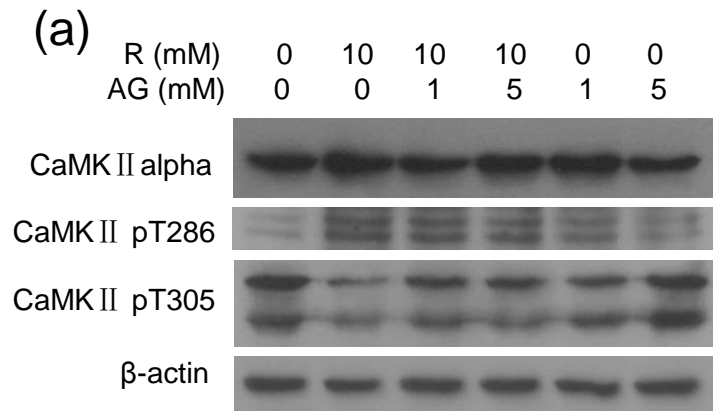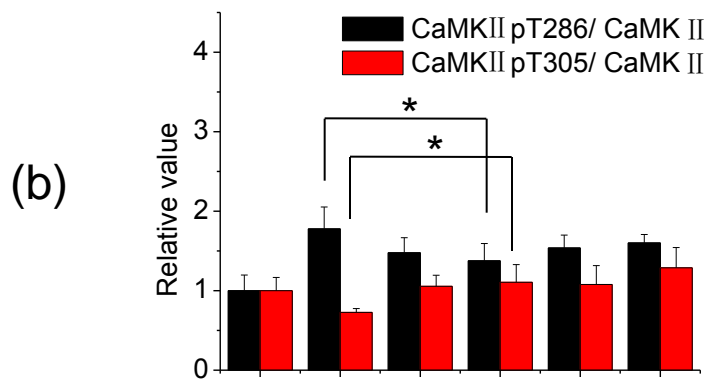

# Supplementary Figure 10

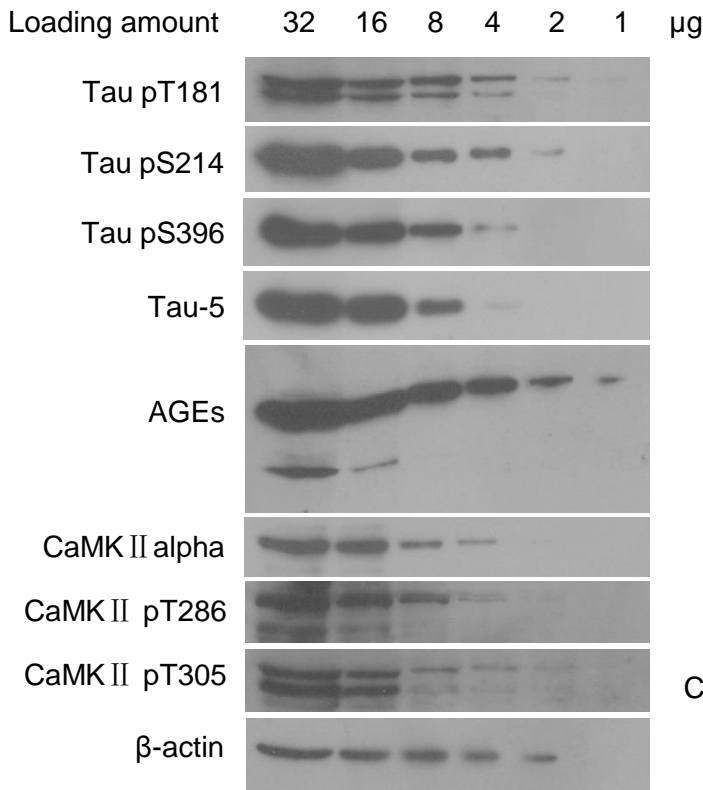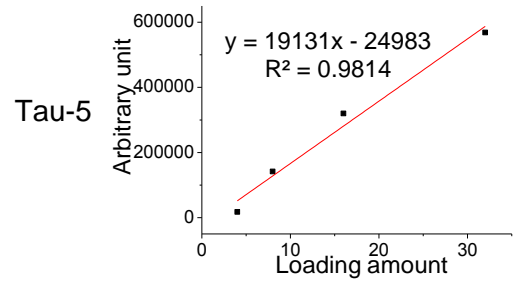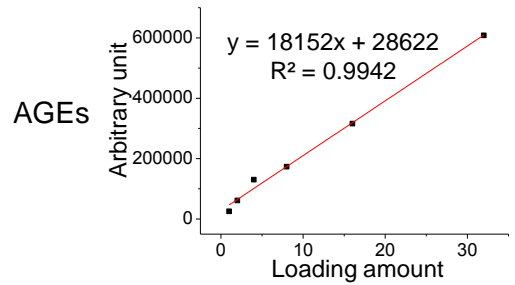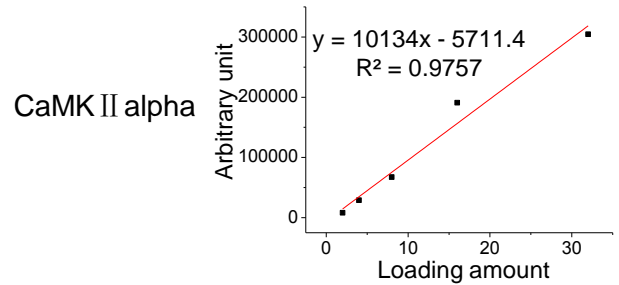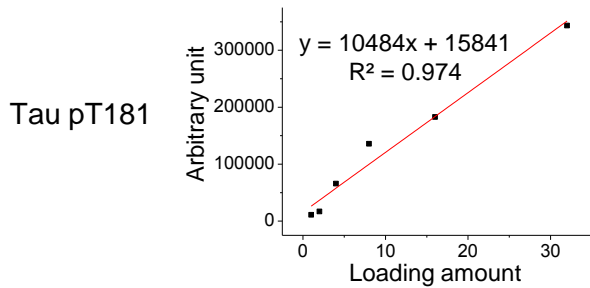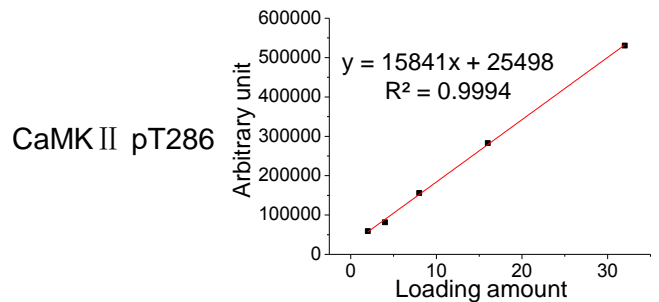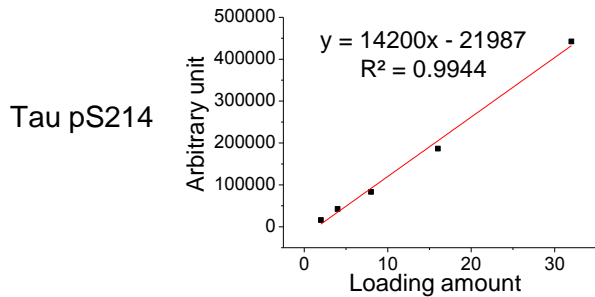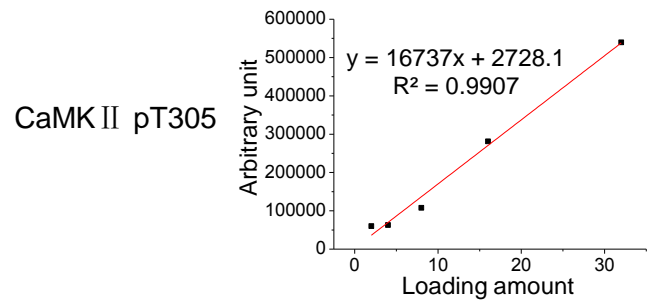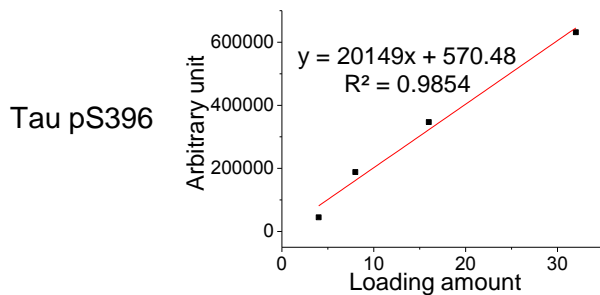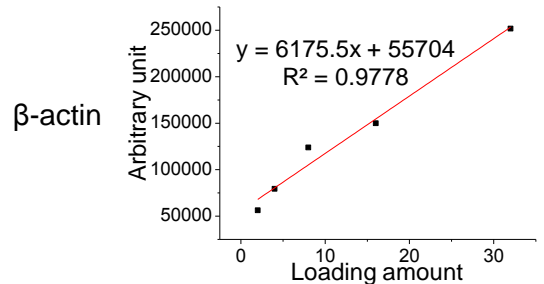

Supplement: Supplementary file 1 [file acel0014-0754-sd1.pdf]
