# Supplementary material for: Ribosylation triggering Alzheimer’s disease-like Tau hyperphosphorylation via activation of CaMKII
Source: Aging Cell. 2015 Jun 11;14(5):754–63. doi: 10.1111/acel.12355 (PMC4568963; doi:10.1111/acel.12355)
Supplement: Supplementary file 2 [file acel0014-0754-sd2.docx]

**Supplementary data**

Supplementary Figure 1. Immunofluorescent staining of AGEs and phosphorylated Tau in the mouse brain.

Supplementary Figure 2. Performance of mice injected with D-glucose in the Morris water maze test and Western blotting of phosphorylated Tau in the mouse brain.

Mice were injected (i.p.) with D-glucose or saline (control) daily for 3 months. The length of time mice took to find the hidden platform was recorded as latency of escape during each of the nine training days (panel a). The percent of the searching distance and time spent in the quadrant where the platform was removed during the probe trial is shown in b. All values are expressed as means±S.E.M. n=10. Tau phosphorylation (anti-pThr181, anti-pSer214 and anti-pSer396), nonphosphorylation (Tau-1, a monoclonal antibody against dephosphorylated Tau protein) and total Tau (Tau-5) were detected by Western blotting (10 µg brain lysate loaded). β-actin was used as a control. AGE formation in the brain and serum was also detected (panel c). n=3.

Supplementary Figure 3. Viability of cells treated with D-glucose.

Conditions were as described for Figure 3c, except N2a cells were incubated with different concentrations of D-glucose.

Supplementary Figure 4. Tau phosphorylation following the treatment of ribosylation in rat primary neurons.

Conditions were as described for Figure 3e, 3e’, except for rat primary neurons were used instead.

Supplementary Figure 5. AGEs formation and Tau phosphorylation following the treatment of D-glucose in N2a cells.

Conditions were as described for Figure 3a,3a’ and 3e,3e’, except D-glucose was used instead of D-ribose.

Supplementary Figure 6. Activity assay of CaMKⅡ.

D-ribose or D-glucose (final concentration 10 mM) was added to N2a cells and incubated for 24 hours. Cells were harvested for CaMKⅡ activity assay by using the SignaTECT® Calcium/Calmodulin-Dependent Protein Kinase Assay System (Promega V8161, USA) (panel a). CaMK2α (0.1 µg/µl, Sigma C9869, USA) was incubated with D-ribose or D-glucose (final concentration 10 mM) for 24 hours at room temperature, followed by CaMKⅡ activity assay (panel b). n=3. Data are expressed as mean ± S.E.M., * denotes P﹤0.05 versus control.

Supplementary Figure 7. Effect of D-ribose on Tau phosphatases in N2a cells following the treatment of D-ribose.

N2a cell lysate extracts were separated by SDS-PAGE and levels of Tau phosphatases were determined using antibodies directed at PP2A, PP2A pTyr307, and PP2A methyl Leu309. Treatment with different concentrations of D-ribose (0, 5, 10, 20, 50, and 100 mM) for 24 hours was shown in panel a,a’, and treatment with D-ribose (10 mM) for 0, 4, 8 ,24, and 48 hours was panel b,b’, respectively. For phospho- or methyl- epitope, relative immunoreactive band intensities are expressed as a ratio to total PP2A. For total PP2A, relative values are expressed as a ratio to actin beta. Data are expressed as mean ± SD.

Supplementary Figure 8. PP2A activity assay.

D-ribose or D-glucose (final concentration 10 mM) was added to N2a cells and incubated for 24 hours. Cells were harvested for PP2A activity assay by using the Serine/Threonine Phosphatase Assay System (Promega V2460, USA). n=4.

Supplementary Figure 9. Effect of aminoguanidine (AG) on CaMKII activity in N2a cells.

Conditions were as described for Figure 5c, except phospho-CaMKII and CaMKII antibodies were used instead.

Supplementary Figure 10. Dilution experiment for representive antibodies.

Different amount of N2a cell lysate was applied for Western blotting, by using the antibodies as indicated. The blots were then adopted for statistical analysis.
